# Testosterone deficiency worsens mitochondrial dysfunction in APP/PS1 mice

**DOI:** 10.3389/fnagi.2024.1390915

**Published:** 2024-05-01

**Authors:** Tianyun Zhang, Yun Chu, Yue Wang, Yu Wang, Jinyang Wang, Xiaoming Ji, Guoliang Zhang, Geming Shi, Rui Cui, Yunxiao Kang

**Affiliations:** ^1^Postdoctoral Research Station of Biology, Hebei Medical University, Shijiazhuang, China; ^2^Laboratory of Neurobiology, Hebei Medical University, Shijiazhuang, China; ^3^Department of Neurology, Third Hospital of Hebei Medical University, Shijiazhuang, China; ^4^Neuroscience Research Center, Hebei Medical University, Shijiazhuang, China

**Keywords:** testosterone deficiency, mitochondrial dysfunction, mitochondrial biogenesis, mitochondrial dynamics, hippocampus, Alzheimer’s disease

## Abstract

**Background:**

Recent studies show testosterone (T) deficiency worsens cognitive impairment in Alzheimer’s disease (AD) patients. Mitochondrial dysfunction, as an early event of AD, is becoming critical hallmark of AD pathogenesis. However, currently, whether T deficiency exacerbates mitochondrial dysfunction of men with AD remains unclear.

**Objective:**

The purpose of this study is to explore the effects of T deficiency on mitochondrial dysfunction of male AD mouse models and its potential mechanisms.

**Methods:**

Alzheimer’s disease animal model with T deficiency was performed by castration to 3-month-old male APP/PS1 mice. Hippocampal mitochondrial function of mice was analyzed by spectrophotometry and flow cytometry. The gene expression levels related to mitochondrial biogenesis and mitochondrial dynamics were determined through quantitative real-time PCR (qPCR) and western blot analysis. SH-SY5Y cells treated with flutamide, T and/or H_2_O_2_ were processed for analyzing the potential mechanisms of T on mitochondrial dysfunction.

**Results:**

Testosterone deficiency significantly aggravated the cognitive deficits and hippocampal pathologic damage of male APP/PS1 mice. These effects were consistent with exacerbated mitochondrial dysfunction by gonadectomy to male APP/PS1 mice, reflected by further increase in oxidative damage and decrease in mitochondrial membrane potential, complex IV activity and ATP levels. More importantly, T deficiency induced the exacerbation of compromised mitochondrial homeostasis in male APP/PS1 mice by exerting detrimental effects on mitochondrial biogenesis and mitochondrial dynamics at mRNA and protein level, leading to more defective mitochondria accumulated in the hippocampus. *In vitro* studies using SH-SY5Y cells validated T’s protective effects on the H_2_O_2_-induced mitochondrial dysfunction, mitochondrial biogenesis impairment, and mitochondrial dynamics imbalance. Administering androgen receptor (AR) antagonist flutamide weakened the beneficial effects of T pretreatment on H_2_O_2_-treated SH-SY5Y cells, demonstrating a critical role of classical AR pathway in maintaining mitochondrial function.

**Conclusion:**

Testosterone deficiency exacerbates hippocampal mitochondrial dysfunction of male APP/PS1 mice by accumulating more defective mitochondria. Thus, appropriate T levels in the early stage of AD might be beneficial in delaying AD pathology by improving mitochondrial biogenesis and mitochondrial dynamics.

## Introduction

Alzheimer’s disease (AD) is the prevalent age-related neurodegenerative dementia, characterized by β-amyloid deposition, pathologic tau and neurodegeneration (Jack et al., 2018). In early AD, mitochondrial dysfunction is already present in afflicted brain regions of numerous AD models and AD patients, such as decrease in oxidative phosphorylation, mitochondrial membrane potential, ATP levels, mitochondria number (Macdonald et al., 2018), as well as increase in reactive oxygen species (ROS) production and oxidative damage (Martins et al., 2018; Butterfield and Halliwell, 2019). Increasing evidence supports the hypothesis that the mitochondrial dysfunction, as an early event, might trigger amyloid beta (Aβ) accumulation and a battery of abnormalities (Swerdlow, 2018), albeit Aβ may instead incite mitochondrial insult. The age-dependent mitochondrial dysfunctions are becoming critical hallmarks of AD pathogenesis.

Reactive oxygen species-induced oxidative stress has an equally important role in the pathologic process of AD (Butterfield and Halliwell, 2019). Normally, adequate antioxidant systems balance the levels of ROS, a by-product of mitochondrial metabolism. However, under pathological conditions, excessive ROS causes severe oxidative stress, showing antioxidant deficiency, and oxidative damage to lipid and protein (Yu, 1994). In early stages of AD, damage to mitochondria induces elevation of ROS production, contributing to the deposition of Aβ and clinical symptoms (Uttara et al., 2009; Guo et al., 2013; Singh et al., 2019). Notably, the decline of the antioxidant levels, such as glutathione (GSH) and superoxide dismutase (SOD), as well as the increase of peroxidation products, such as malondialdehyde (MDA) and 3-nitrotyrosine (3-NT), is observed in AD progression (Beal, 2002; Padurariu et al., 2010; Cacciatore et al., 2012). Thus, attenuating oxidative stress through improving mitochondrial function is beneficial for reducing AD incidence.

Testosterone (T) levels in elderly men decrease with age. Low T levels in older male individuals are closely related to the development of cognitive impairment (Dong et al., 2021) and the aggravated AD symptoms are found in male AD patients with decreased T levels (Muller et al., 2008). Research conducted on adult male rats subjected to gonadectomy-induced T deficiency demonstrates the attenuated brain mitochondrial function and the increased oxidative damage (Meydan et al., 2010; Hioki et al., 2014; Yan et al., 2017). Conversely, T supplementation to aged male rats improves mitochondrial function and the enhanced antioxidative capacity within their brains (Yan et al., 2021). Age-related decline of T levels seems one of risk factors for AD pathogenesis in elderly men (Yeap and Flicker, 2022), while mitochondria might be that potential target affected by this risk factor in the development of AD. Consequently, in this study, male APP/PS1 mutant transgenic mice were utilized to assess the altered cognitive abilities, mitochondrial functions, and critical factors involved in regulating mitochondrial function (mitochondrial biogenesis and mitochondrial dynamics) in T-deficiency AD mouse models. Furthermore, to explore the mechanisms underlying T deficiency’s effects on mitochondrial function in male APP/PS1 mutant transgenic mice, the effects of T on mitochondrial biogenesis and mitochondrial dynamics were also investigated in human SH-SY5Y cells. This *in vitro* study focused on detecting the expression alterations of genes involved in mitochondrial biogenesis and dynamics under oxidative stress following androgen receptor (AR) inhibition.

## Materials and methods

### Animals and treatments

**FIGURE 1:**
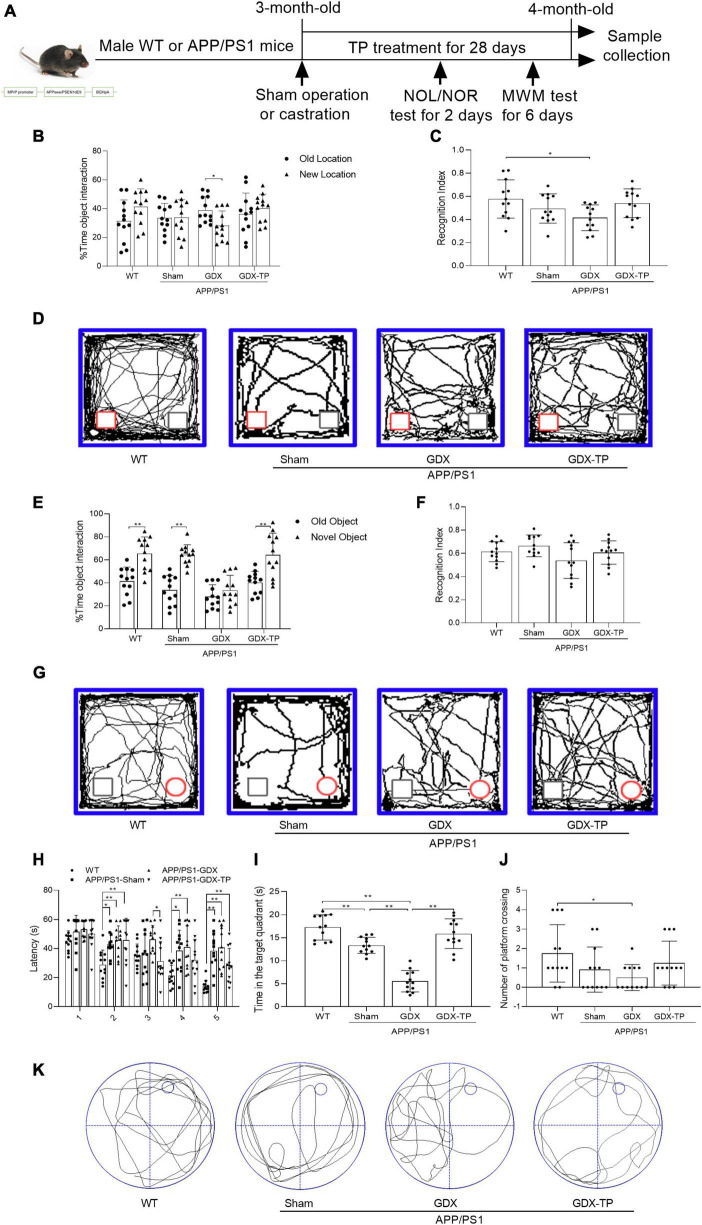
Effects of testosterone deficiency on cognitive-behavioral deficits in male APP/PS1 mice. **(A)** The gene modification map is shown, and the research process is illustrated, including sham operation or castration, TP treatment and behavioral tests. A novel object location (NOL) test was performed to analyze the location memory of the mice. It included a familiarization trial, 1 h retention interval and a novel location test. **(B)** The % time object interaction and **(C)** recognition index (calculated as the ratio of time spent exploring the new location to total exploration time) of each mouse were compared. **(D)** Representative moving traces in NOL test show the exploration to the familiar (gray square) and the novel (red square) location. A novel object recognition (NOR) test was performed to analyze the object recognition memory of the mice. It included a familiarization trial, 1 h retention interval and a novel object test. **(E)** The % time object interaction and **(F)** recognition index (calculated as the ratio of time spent exploring the novel object to total exploration time) of each mouse were compared. **(G)** Representative moving patterns in NOR test show the exploration to the familiar (gray square) and the novel (red circle) object. Morris water maze (MWM) test was performed to assess the learning and spatial memory of the mice. **(H)** Escape latency, **(I)** time in the target quadrant, **(J)** number of platform crossing, and **(K)** the representative path patterns were tested. WT, wild-type; TP, testosterone propionate. Data are expressed as the mean ± SD (*n* = 12). *P < 0.05 and **P < 0.01.

Male APPswe/PSEN1dE9 double transgenic mice overexpressing the mutated genes for human amyloid precursor protein and presenilin 1 (APP/PS1; Figure 1A), as well as age-matched male wild-type (WT) mice were purchased from Beijing HFK Bioscience Co., China. The mice were provided food and water *ad libitum* and housed under controlled conditions (22 ± 2°C and 12 h/12 h light–dark cycle). All the animal experiments were conducted in accordance with the “Guidelines for the Care and Use of Mammals in Neuroscience and Behavioral Research,” and were approved by the Laboratory Animal Ethical and Welfare Committee of Hebei Medical University (IACUC-Hebmu-2023054). The experimental mice were divided into following four groups: WT group, APP/PS1-Sham group, gonadectomized APP/PS1 (APP/PS1-GDX) group, and gonadectomized APP/PS1 with T propionate (TP) supplementation (APP/PS1-GDX-TP) group, where mice were subjected to either bilateral orchiectomy or sham operation. For APP/PS1-GDX-TP mice, male APP/PS1 mice were castrated, and subcutaneously injected immediately with TP at 1 mg/kg per day for 28 days. This dosage was adopted based on a previous study (Qiu et al., 2016). All the mice were sacrificed at the age of 4 months. A previous study illustrated that APP/PS1 mice show mild cognitive deficits at this age (Yu et al., 2019). The experimental procedures were briefly illustrated in Figure 1A.

### Object location test and object recognition test

On the 8th day before sacrifice, mice underwent habituation in a 40 cm by 40 cm soundproof square open field for 5 min. The next day, a novelty recognition test was conducted using the same apparatus. The test consisted of a familiarization trial, novel object localization (NOL) test, and novel object recognition (NOR) test. The experimental procedure followed a previously established method, with minor modifications from the study (Jacobs et al., 2021). In brief, the mice were exposed to two identical wooden objects during the 10-min familiarization trial. After a 1-h interval, the NOL test began, where one object was relocated. Another 1-h interval followed, and the mice were subjected to the NOR test, where one object was replaced with a novel hemisphere-shaped wooden object. The DigBehv Animal Behavior Analysis Software was used to record the time mice spent exploring each object within 2 cm and calculate exploration ratios and recognition indices. The objects and arena were cleaned with 70% ethanol between each trial.

### Morris water maze test

During the last 6 days prior to their sacrifice, the mice were subjected to water maze test to evaluate their spatial learning and memory abilities. These tests were conducted in a controlled environment within a soundproof room. The main apparatus used for the experiment was a circular plastic tank with a diameter of 120 cm and height of 50 cm, which was partially filled with opaque water. A platform with a diameter of 9 cm was concealed 1 cm beneath the water’s surface, located in the first quadrant and served as an escape point for the mice during the trials. The training sessions were conducted over the first 5 days. Each daily session consisted of four trials, with an hour interval between each trial. In each trial, the mouse was placed in the water maze starting from different quadrants each time, and was given 60 s to navigate the maze and locate the hidden platform. The time taken to reach the platform, known as the escape latency, was recorded. On the sixth day, a probe session was conducted. During this session, the escape platform was removed from the maze. Each mouse was given 60 s to freely swim in the maze. The time spent swimming in the first quadrant, as well as the number of crossings over the platform’s previous position, were recorded. To accurately analyze the movement patterns of the mice during the trials, the DigBehv Animal Behavior Analysis Software (Shanghai Jiliang Software Technology Co., China) was utilized.

### Sample preparation

For biochemical or molecular biological assays, mice were sacrificed by decapitation and brains were removed quickly. Bilateral hippocampi were dissected, which were immediately used for biochemical assays or frozen in liquid nitrogen and stored at −80°C for later use based on the study aims. After decapitation, seminal vesicles were dissected, blotted and weighed. Trunk blood was collected and serum was harvested by centrifugation and stored at −80°C for measuring T levels. For immunohistochemistry (IHC) staining studies, mice were anesthetized and perfused transcardially with 4% paraformaldehyde in 0.1 M phosphate buffer (pH 7.4). For mitochondrial ultrastructure analysis, mice were perfused transcardially with fixative containing 2% paraformaldehyde and 1.25% glutaraldehyde. Brains were postfixed in the corresponding fixative for 24 h at 4°C.

### Immunohistochemistry

The postfixed brain tissues were dehydrated in graded ethanol, cleared in xylene, and embedded in paraffin wax. Wax-embedded brain blocks were sliced into 5 μm coronal sections. The sections were dewaxed using xylene and gradient ethanol for deparaffinization and rehydration. The brain sections were blocked in 5% normal goat serum after antigen retrieval, and then incubated with rabbit anti-Aβ_1–42_ (Biolegend, specifically recognizing extracellular Aβ_1–42_) or mouse anti-3-NT antibody (Santa Cruz Biotechnology) overnight at 4°C. After three washes in phosphate buffer, the sections were incubated with goat anti-rabbit IgM-HRP or goat anti-mouse IgG-HRP for 2 h at room temperature. All sections were stained for 5 min in liquor containing 0.05% diaminobenzidine and 0.03% H_2_O_2_ in 0.05 M Tris–HCl buffer (pH 7.6). The photograph of stained sections was taken by light microscope (Olympus). The area/number of the Aβ_1–42_ plaques and the average optical density of 3-NT were measured by Image Pro Plus 6.0.

### Spectrophotometry analysis

For spectrophotometry analysis, hippocampal tissue blocks were weighed and homogenized immediately with 0.01 M ice-cold phosphate buffer saline (PBS, pH 7.4) and centrifuged based on the study purposes. Levels of MDA, the ratio of GSH/oxidized GSH (GSH/GSSG), the activities of CuZn-SOD and Mn-SOD, ATP levels, as well as the activities of mitochondrial complexes and citrate synthase (CS) were measured spectrophotometrically according to the instructions of the corresponding detection kits (MDA: Code A003-1; GSH/GSSG: Code A061-1; CuZn-SOD and Mn-SOD: Code A001-2, Nanjing Jiancheng Bioengineering Institute, China; ATP: AKOP004M; Complex I: AKOP005M; Complex II: AKOP006M; Complex III: AKOP007M; Complex IV: AKOP008M; Complex V: AKOP009M; and CS: AKAC007M, Boxbio, China). Serum T levels were measured using a T ELISA kit (Item No. 582701, Cayman) based on the kit instruction. For spectrophotometry analysis, SH-SY5Y cells seeded in 96-well plates (4 × 10^4^ cells/well) were processed by different treatments. The cell viability after different treatments was detected with MTT method by adding 50 μl DMEM/F12 and 50 μl MTT reagents to the wells. After incubation for 3 h at 37°C, 150 μl of DMSO was added to the wells and their absorbance was read at a wavelength of 590 nm. The cell GSH/GSSG ratio and ATP levels were respectively measured using the GSH and GSSG detection kits (S0053, Beyotime Institute of Biotechnology, China) and the ATP levels detection kit (A095-1-1, Nanjing Jiancheng Bioengineering Institute, China), with the absorbance read at 412 nm for GSH and GSSG and at 636 nm for ATP levels. The levels were normalized to the total protein amount of the sample.

### Mitochondrial membrane potential detection

Mitochondrial membrane potential was estimated in dissociated hippocampal cells using JC-1 dye that has two forms of fluorescence (Cossarizza et al., 2019). The ratio of aggregate form (exhibiting red fluorescence) to monomer form (exhibiting green fluorescence) reflects the level of mitochondrial membrane potential. Tissue blocks were grinded in PBS solution and filtered using a nylon mesh screen. The harvested cells were incubated in JC-1 solution (10 μg/ml, Cayman) at 37°C for 30 min, washed, resuspended in 500 μl PBS, and immediately analyzed by flow cytometry. The red fluorescence intensity was measured at Ex/Em: 525/590 nm. The green fluorescence intensity was measured at Ex/Em: 490/530 nm. The ratio of JC-1 aggregates to JC-1 monomers was calculated.

### Mitochondrial ultrastructure analysis

The hippocampal tissue blocks were dissected and washed in phosphate buffer, they were postfixed with 1% osmium tetroxide for 2 h, dehydrated in acetone, and embedded in araldite. Ultrathin sections (70 nm) were obtained with a microtome. After staining with uranyl acetate and lead citrate, the sections were examined by a transmission electron microscope (Hitachi HT7800) at 80 kV for mitochondrial ultrastructural alteration. The abnormal mitochondria rate was calculated by the number of abnormal mitochondria/total number of mitochondria (Mansueto et al., 2017).

### Cell culture and treatment

Human neuroblastoma SH-SY5Y cells were used in *in vitro* studies (ATCC, Manassas, VA, USA). They were grown in DMEM/F12 medium containing 10% fetal bovine serum and 1% penicillin and streptomycin at 37°C in a humidified incubator with 5% CO_2_ and 95% air flow. For cell viability experiments, the cells were first treated by T (1, 10, and 100 nM) for 24 h and then by H_2_O_2_ (0, 50, 100, 200, and 300 μM) for 24 h (Grimm et al., 2014). Flutamide (F, AR receptor antagonist) pretreatment for 1 h was chosen to test the role of AR in the effects of T on cell viability in exposure to H_2_O_2_. For the experiment about the effects of AR inhibitor and T on the oxidative damage, SH-SY5Y cells were treated with F for 1 h, and treated with T for 24 h, followed with H_2_O_2_ treatment for 24 h. In this part, four groups were included, that were, control group, H_2_O_2_ group, T + H_2_O_2_ group, F + T + H_2_O_2_ group. Stock solutions of T and F were prepared in DMSO (final concentration of DMSO was 0.001%). Stock solution of H_2_O_2_ was prepared in DMEM/F12. The control group treated with vehicle solutions (DMSO or DMEM/F12). Cells were plated for treatment when they reached 70%–80% confluence. After treatment, cells were washed twice with cold PBS, then were subjected to corresponding detection.

### Mitochondrial stress testing

Oxygen consumption rate (OCR) of cells was examined using a Seahorse Bioscience XF24 Analyzer. Cells were seeded in XF24 cell culture microplate at a density of 2.5 × 10^4^ cells/well. After the cells adhered to the wall, culture medium was replaced with medium containing 1 mM sodium pyruvate, 2 mM glutamine, and 10 mM glucose, Then, the microplate was placed in a CO_2_-free incubator at 37°C for 1 h. The OCR was recorded simultaneously before and after the sequential injection of oligomycin (0.5 μM), FCCP (2 μM), and rotenone and antimycin A (0.5 μM). Data were extracted from Seahorse XF24 software, and were normalized to cell counts.

### Cell fluorescence staining

Cells were cultured on glass slides. After treatment, JC-1 working solution (Item No. 15003, Cayman, USA, 10 μg/ml), MitoTracker Red CMXRos (M7512, Invitrogen, USA), and Hoechst 33258 (Beyotime Institute of Biotechnology, China) were added in the culture medium respectively according to different experimental purposes. After incubation in dark at 37°C for 10 min, the cells were washed in warm PBS gently. Fluorescent images were acquired using a two-photon microscope (FV1200MPE, OLYMPUS). JC-1 monomers were detected at Ex/Em 490/530 nm, JC-1 aggregates were detected at Ex/Em 525/590 nm, mitochondria stained with MitoTracker Red CMXRos were detected at Ex/Em 579/599 nm, and cells stained with Hoechst 33258 were detected at Ex/Em 530/461 nm. Ten fields were randomly selected and at least 100 cells were analyzed in each sample. The mean fluorescence intensity (integrated density/area of view) of each cell was measured using ImageJ software (Pediconi et al., 2023). Mitochondrial membrane potential was analyzed by calculating the ratio of red to green fluorescence. The mitochondrial mass was expressed by the mean fluorescence intensity of mitochondria stained with MitoTracker Red (Sghaier et al., 2019). Punctiform mitochondria were considered fragmented mitochondria, mitochondria with clear networks were considered tubular mitochondria. The number of cells was counted when 80% of mitochondria in the cell are under mitochondrial fragmentation.

### Quantitative real-time PCR analysis

After extraction with TRIzol from hippocampal tissue blocks or cultured cells, 1 μg of total RNA was reverse-transcribed using random primers to obtain the first-strand cDNA template. Then, quantitative real-time PCR (qPCR) was performed with 2 μl of cDNA (diluted 1:10), 2 μl of each specific primer, and 2 × All-in-One™ qPCR Mix (GeneCopoeia Inc.) in a final volume of 20 μl. PCR was performed as follows: an initial cycle at 95°C for 15 min, followed by 40 cycles at 95°C for 10 s, 60°C for 20 s, and 72°C for 20 s. The melting curves of the PCR products were analyzed to confirm the specificity of amplification. Gene expression was analyzed using glyceraldehyde-3-phosphate dehydrogenase (GAPDH) as internal control. For all samples, qPCR was performed in triplicate. Relative quantification was performed using the 2^–ΔΔCt^ method. The sets of primers were as follows: for mouse genes, *SYP* (5′-cag ttccgggtggtcaagg-3′ and 5′-actctccgtcttgttggcac-3′), *PSD-95* (5′-taccaaagaccgtgccaacg-3′ and 5′-cggcattggctgagacatca-3′), *Mn-SOD* (5′-tgaacaacctcaacgccac-3′ and 5′-gaaggtagtaagcgtg ctc-3′), *PGC-1*α (5′-gaaagggccaaacagagaga-3′ and 5′-gta aatcacacggcgctctt-3′), *NRF-1* (5′-tggagtccaagatgctaatg-3′ and 5′-agagctccatgctactgttc-3′), *TFAM* (5′-caggaggcaaaggatgattc-3′ and 5′-ccaagacttcatttcattgtcg-3′), *Drp1* (5′-caggaattgttacggttcccta-3′ and 5′-cctgaattaacttgtcccgtg-3′), *Mfn1* (5′-aacttgatcgaatagcatccga-3′ and 5′-gcattgcattgatgacagag-3′), *OPA1* (5′-gatgacacgctctccagtg-3′ and 5′-tcggggctaacagtacaa-3′), *GAPDH* (5′-actcttccaccttcgatgcc-3′ and 5′-tcttgctcagtgtccttgct-3′); for human genes, *PGC-1*α (5′-ctg caggcctaactccaccca-3′ and 5′-actcggattgctccggccct-3′), *NRF-1* (5′-ctactcgtgtgggacagcaa-3′ and 5′-aattccgtcgatggtgagag-3′), *TFAM* (5′-ctactcgtgtgggacagcaa-3′ and 5′-aattccgtcgatggtgagag-3′), *Drp1* (5′-aatcgtcgtagtgggaacgc-3′ and 5′-tccaccccattttcttctcct-3′), *Mfn1* (5′-gcctcctctccgcctttaactt-3′ and 5′-gccttcttagccagcacaaag-3′), *OPA1* (5′-cgggaacttgaccggaatga-3′ and 5′-cgcagctggaaggtagatgt-3′), and *GAPDH* (5′-ctcctccacctttgacgctg-3′ and 5′-ccacc ctgttgctgtagcca-3′).

### Mitochondrial DNA copy number detection

For mice experiment, total DNA was extracted from tissue blocks using an animal tissue genomic DNA kit (ZP307-2, ZOMANBIO), while it was extracted from SH-SY5Y cells by cell genomic DNA extraction kit (ZP308, ZOMANBIO). Mitochondrial DNA (mtDNA) copy number was determined by quantifying mitochondrial gene (16S rRNA for mice and D-Loop for human cells) and nuclear-encoded gene (HK2 for mice and Actin for human cells) expression via qPCR. The sets of primers were as follows: for mouse genes, *16S rRNA* (5′-ccgcaagggaaagatgaaagac-3′ and 5′-tcgtttggtttcggggtttc-3′), *HK2* (5′-gccagcctctcctgattttagtgt-3′ and 5′-gggaacacaaaagacctcttctgg-3′); for human genes, *D-Loop* (5′-accacccaagtattgactcaccca-3′ and 5′-ccgtacaatattcatggtggctggc-3′), *Actin* (5′-cccctggcggcctaaggact-3′ and 5′-acatgccggagccgttgtcg-3′). qPCR was performed in triplicate. Relative mtDNA copy number was calculated by the ratio of mitochondrial gene to nuclear-encoded gene using the 2^–ΔΔCt^ method.

### Western blot analysis

The proteins from hippocampal tissue blocks or SH-SY5Y cells were homogenized in ice-cold RIPA buffer (No. R0010, Solarbio, China) containing 1% PMSF. An immunoblotting analysis was performed following a previous method (Yan et al., 2021). The presence of particular proteins was examined using rabbit anti-synaptophysin (SYP, Abways), rabbit anti-postsynaptic density protein 95 (PSD-95, Abways), rabbit anti-Mn-SOD (HuaBio), rabbit anti-peroxisome proliferator-activated receptor-γ coactivator 1αantibody (PGC-1α, ABclonal), rabbit anti- nuclear respiratory factor 1 antibody (NRF-1, ABclonal), rabbit anti- mitochondrial transcription factor antibody (TFAM, GeneTex), rabbit anti-mitochondrial fission protein dynamin-related protein (Drp1, Cell Signaling Technology), rabbit anti-mitochondrial fusion protein mitofusin 1 (Mfn1, arigo), rabbit anti-optic atrophy protein 1 (OPA1, GeneTex), or mouse anti-3-NT antibody (Santa Cruz Biotechnology). After washing three times, the membrane was incubated for 2 h in IRDye^®^ 800-conjugated goat anti-rabbit secondary antibody (Rockland) or goat anti-mouse secondary antibody (Rockland) at room temperature. The relative band densities were measured by an Odyssey infrared scanner (LICOR Biosciences, USA). The expression of target proteins was normalized to β-actin that was used as endogenous control (rabbit anti-β-actin antibody, Santa Cruz Biotechnology).

### Statistical analysis

Data are shown as the mean ± standard deviation (SD). Grubb’s test was applied to remove possible outliers. Levene’s test was applied to test the homogeneity of variance. If homogeneity of variance is equal, statistical analysis was applied by one-way analysis of variance (ANOVA, *F*-statistic) followed by Tukey’s multiple comparison test. If the variance was unequal, Welch’s *F* test in one-way ANOVA (*F*′-statistic) was used and *post hoc* analyses were done using the Games-Howell procedure. For the Morris maze, escape latency was analyzed using two-way ANOVA with repeated measures, and the number of platform crossings was tested using Chi-square test. Statistical analyses were performed using the SPSS 21 software. *P* < 0.05 was considered statistically significant.

## Results

### Testosterone deficiency aggravated the cognitive deficits of male APP/PS1 mice

In male AD model mice, gonadectomy was performed to induce androgen depletion, which is verified by significantly reduced seminal vesicle weight (seminal vesicle weight/body weight: 0.6 × 10^–3^ ± 0.4 × 10^–3^, a sensitive bioassay of androgen levels; Yamane et al., 1986) and serum T levels (0.1 ± 0 ng/ml), compared with sham-operated APP/PS1 mice (seminal vesicle weight/body weight: 5.1 × 10^–3^ ± 0.8 × 10^–3^, serum T levels: 7.4 ± 1.0 ng/ml). As a control for APP/PS1-GDX mice, the cognitive, pathological, and mitochondrial changes of APP/PS1-Sham mice were firstly compared with WT mice fully. To explore effects of T deficiency on the cognitive performance of the 4-month-old APP/PS1 mice, NOL (Figures 1B–D), NOR (Figures 1E–G), and Morris water maze (MWM) (Figures 1H–K) tests were performed. NOL and NOR test are sensitive to detect impaired recognition memory (Leger et al., 2013). Considering the GDX-induced apathy and motor problem may affect the interactions of experimental animals with the objects in NOL and NOR tests, MWM test were performed following NOL and NOR test. Morris water maze test is common methods to evaluate hippocampal-dependent learning, acquisition of spatial memory and long-term spatial memory (Jacobs et al., 2021). In NOL test, all the mice spent the same time in exploring the old and the novel localization of the familiar objects, except for the APP/PS1-GDX mice that spent less time on exploration in the new location than that in the old location (Figure 1B). APP/PS1-GDX mice showed significantly low recognition index compared with WT mice (Figure 1C). In NOR test, the mice in WT, APP/PS1-Sham, and APP/PS1-GDX-TP groups exhibited more time exploring the novel object and APP/PS1-GDX mice spent similar time exploring either the new or the old object (Figure 1E). In Morris water maze test, during the 5 training days, the escape latency of WT mice progressively decreased, while APP/PS1 mice with or without castration exhibited longer escape latency than WT mice in the 2nd, 4th and 5th day (Figure 1H). An obviously decrease of escape latency was found in APP/PS1-GDX-TP mice compare with APP/PS1-GDX mice in the 3rd training day, and a slight but non-significant reduction of escape latency was observed in APP/PS1-GDX-TP mice, when compared with APP/PS1-Sham mice and APP/PS1-GDX mice in the last 2 days. In the probe test, group difference was found in the time spent in the target quadrant and in the number of platform crossing (Figures 1I–K). APP/PS1-Sham mice spent less time in the target quadrant than WT mice and orchiectomy further decreased time spent of APP/PS1 mice in the target quadrant (Figure 1I). TP supplementation to APP/PS1-GDX mice restored the time spent in the target quadrant to the level of APP/PS1-Sham mice as well as WT mice. Relative to WT mice, APP/PS1-GDX mice showed the reduced number of platform crossing (Figure 1J). Testosterone deficiency further compromises the cognitive deficits of male APP/PS1 mice.

### Testosterone deficiency induces pathological alterations of hippocampus in male APP/PS1 mice

**FIGURE 2:**
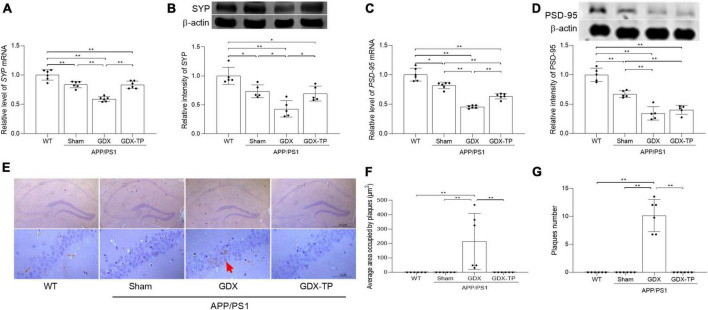
Effects of testosterone deficiency on pathological alterations in the hippocampus of male APP/PS1 mice. qPCR and Western blot were performed respectively to analyze relative **(A)** mRNA levels of *SYP*, **(B)** representative blots for SYP and relative protein levels of SYP, **(C)** relative mRNA levels of *PSD-95*, and **(D)** representative blots for PSD-95 and relative protein levels of PSD-95. The complete Western blots for each sample are provided in Supplementary Data. **(E)** Immunohistochemistry staining revealed the expression of Aβ_1–42_ in hippocampus. **(F)** The area of Aβ_1–42_ plaques. **(G)** The number of Aβ_1–42_ plaques. Data are expressed as the mean ± SD (*n* = 6 for qPCR, *n* = 5 for Western blot, *n* = 6 for immunohistochemistry). *P < 0.05 and **P < 0.01.

The pathological changes of hippocampus in experimental animals were revealed by observing the altered expression of synapse-related proteins (PSD-95 and SYP) as well as Aβ deposition. Significantly decreased expression of PSD-95 and SYP at mRNA and protein levels were detected in the hippocampus of APP/PS1-Sham mice relative to WT mice, which were further reduced in APP/PS1-GDX mice (Figures 2A–D). Significantly increased area and number of Aβ_1–42_ plaques were observed in APP/PS1-GDX mice, compared with mice in other three groups (Figures 2E–G). Testosterone deficiency further increases the pathological alterations of the hippocampus in APP/PS1 mice.

### Testosterone deficiency aggravates oxidative stress in the hippocampus of male APP/PS1 mice

**FIGURE 3:**
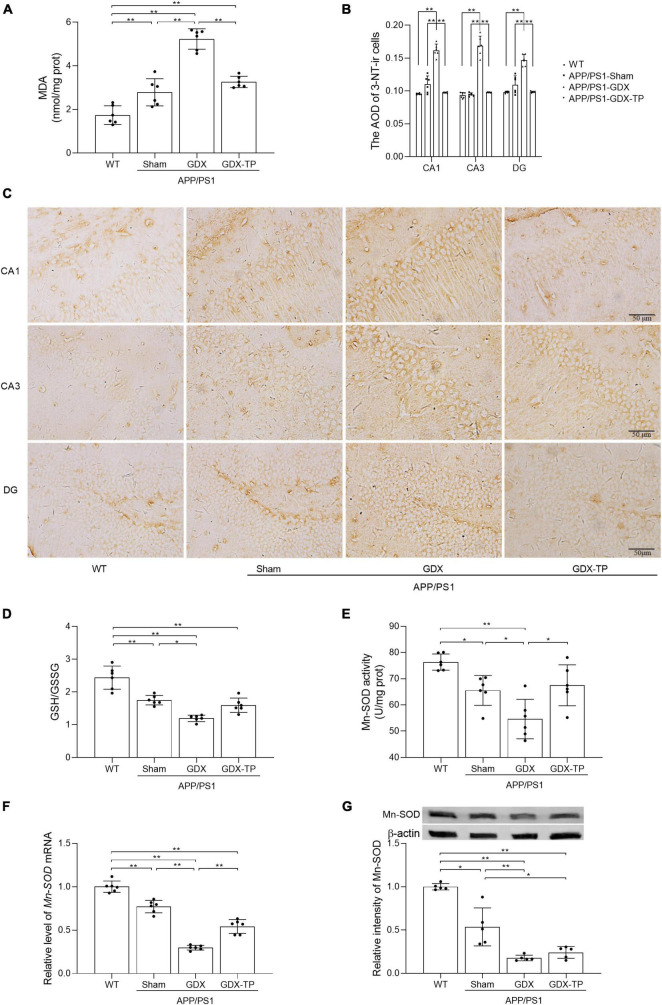
Effects of testosterone deficiency on oxidative stress in the hippocampus of male APP/PS1 mice. **(A)** MDA levels were analyzed by spectrophotometry. **(B,C)** 3-NT, a biomarker of protein nitration and oxidative stress, was detected by immunohistochemistry. Spectrophotometry was also utilized to measure **(D)** the ratio of GSH/GSSG and **(E)** Mn-SOD activity. **(F)** qPCR was used to assess the relative mRNA levels of Mn-SOD. **(G)** Western blot was performed to evaluate the relative protein levels of Mn-SOD, and representative blots for Mn-SOD was shown. The complete representative Western blots for each sample are provided in Supplementary Data. AOD, average optical density. Data are expressed as the mean ± SD (*n* = 6 for spectrophotometry and qPCR, *n* = 6 for immunohistochemistry, *n* = 5 for Western blot). *P < 0.05 and **P < 0.01.

Increased oxidative stress is common in normal aging and AD brain. Oxidative stress is caused by imbalance between production of ROS and antioxidative defense. To assess the extent of oxidative stress, we measured MDA, 3-NT, and GSH/GSSG ratio, as well as CuZn-SOD and Mn-SOD activity/levels. MDA is an important indicator of lipid peroxidation caused by ROS damage to cytoplasmic membrane (Liu et al., 2021). 3-NT is another oxidative damage marker formed due to nitration of protein-bound and free tyrosine residues by reactive peroxynitrite molecules. GSH and GSSG are two different forms of glutathione. As a cofactor for antioxidative enzymes, GSH can be oxidized to GSSG. CuZn-SOD functions in the nucleus and cytoplasm to eliminate ROS, while Mn-SOD works within the mitochondria to clear ROS (Liu et al., 2021). The MDA levels in the hippocampus were significantly higher in male APP/PS1-Sham mice than in WT mice. Testosterone deficiency further increased MDA levels and 3-NT immunoreactive intensity in the hippocampus of APP/PS1 mice (Figures 3A–C). The ratio of GSH/GSSG, as well as the activity and levels of Mn-SOD enzymes in the hippocampus was less in APP/PS1-Sham mice than WT mice (Figures 3D–G). Castration to male APP/PS1 mice further decreased the ratio of GSH/GSSG, as well as the activity and levels of Mn-SOD enzymes in the hippocampus. There was no obvious difference in CuZn-SOD activity between APP/PS1-GDX mice and APP/PS1-Sham mice (Data not shown), though its activity in APP/PS1-Sham mice was significantly low relative to WT mice. Testosterone deficiency aggravates oxidative stress in the hippocampus of male APP/PS1 mice.

### Testosterone deficiency exacerbates mitochondrial dysfunction in the hippocampus of male APP/PS1 mice

**FIGURE 4:**
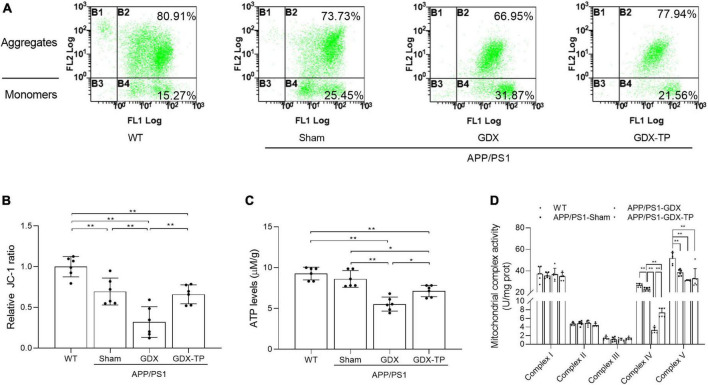
Effects of testosterone deficiency on mitochondrial dysfunction in the hippocampus of male APP/PS1 mice. **(A,B)** Mitochondrial membrane potential was detected by flow cytometry. **(C)** ATP levels and **(D)** mitochondrial complex activities were assessed by spectrophotometry. Data are expressed as the mean ± SD (*n* = 6). *P < 0.05 and **P < 0.01.

Mitochondria are the main source of energy and ROS production. Mitochondrial dysfunction can lead to ROS overproduction and subsequent oxidative stress (Guo et al., 2022). In AD, impairment of mitochondrial functions has been implicated as the primary cause of ROS generation (Tobore, 2019) and is one of the early events of AD. Thus, mitochondrial functions index, such as mitochondrial membrane potential (revealed by the ratio of JC-1 aggregates to monomers, Figures 4A, B), ATP levels (Figure 4C) and the mitochondrial complex activities (Figure 4D) were analyzed, to elucidate whether T deficiency aggravated the mitochondrial dysfunction in the hippocampus of male APP/PS1 mice. Group difference among WT, APP/PS1-Sham, APP/PS1-GDX, and APP/PS1-GDX-TP mice was found in the hippocampal JC-1 ratio and ATP levels, as well as activities of mitochondrial complexes IV and V. APP/PS1-Sham mice showed decreased JC-1 ratio and activities of mitochondrial complexes IV and V than WT mice (Figures 4A, B, D). Testosterone deficiency significantly reduced JC-1 ratio, ATP levels and mitochondrial complex IV activity in the hippocampus of male APP/PS1 mice. There is no group difference in ATP levels between WT mice and APP/PS1-Sham mice. No altered mitochondrial complex activities were observed in complexes I, II, and III among WT, APP/PS1-Sham, APP/PS1-GDX, and APP/PS1-GDX-TP mice, as well as in complex V among APP/PS1-Sham, APP/PS1-GDX, and APP/PS1-GDX-TP mice. Testosterone deficiency exacerbates mitochondrial dysfunction in the hippocampus of male APP/PS1 mice.

### Testosterone deficiency further attenuates mitochondrial biogenesis in the hippocampus of male APP/PS1 mice

**FIGURE 5:**
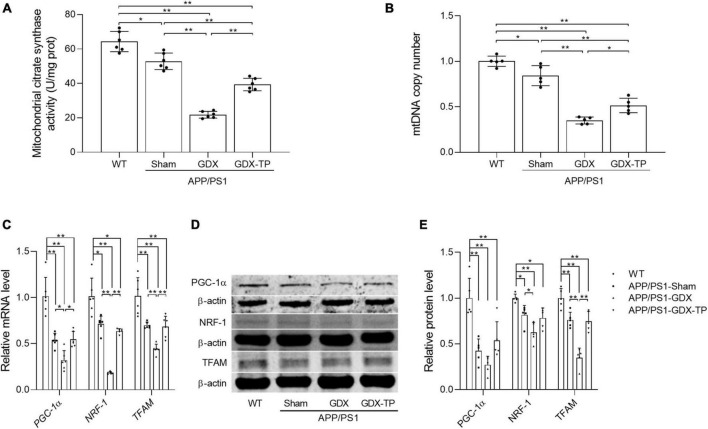
Effects of testosterone deficiency on mitochondrial biogenesis in the hippocampus of male APP/PS1 mice. **(A)** Mitochondrial citrate synthase activity was assessed by spectrophotometry. **(B)** mtDNA copy number was detected by qPCR. **(C)** Relative mRNA levels of *PGC-1*α, *NRF-1*, and *TFAM* were detected by qPCR. **(D)** Representative blots for PGC-1α, NRF-1, and TFAM; **(E)** relative protein levels of PGC-1α, NRF-1, and TFAM were analyzed by Western blot. The complete Western blots for each sample are provided in Supplementary Data. Data are expressed as the mean ± SD (*n* = 6 for spectrophotometry; *n* = 5–6 for qPCR, *n* = 5 for Western blot). *P < 0.05 and **P < 0.01.

Mitochondria are energy source of cells. Local energy deficits are correlated with the reduced content of mitochondria with normal function in afflicted brain area. Mitochondrial biogenesis is a predominant strategy to maintain healthy mitochondrial content. Thus, relevant parameters reflecting mitochondrial content, such as CS activity (a mitochondrial matrix enzyme) and mtDNA copy number, as well as the key inducer and effectors regulating mitochondrial biogenesis, namely PGC-1α, NRF-1, and TFAM (Kozhukhar and Alexeyev, 2022), were analyzed to evaluate the influence of T deficiency upon mitochondrial biogenesis in hippocampus of male APP/PS1 mice. CS activity and mtDNA copy number were significantly reduced in the hippocampus of APP/PS1-GDX mice relative to APP/PS1-Sham mice, as well as WT mice (Figures 5A, B). Decreased CS and mtDNA copy number were also present in APP/PS1-Sham mice compared with WT Mice. Testosterone supplementation to APP/PS1-GDX mice showed amelioration to relevant parameters observed above in hippocampus. In addition, the expression levels of PGC-1α, NRF-1, and TFAM were significantly decreased in APP/PS1-Sham mice relative to WT mice and gonadectomy to male APP/PS1 mice induced further reduction of them (except PGC-1α protein level), which was reversed by T supplementation to APP/PS1-Sham level (Figures 5C–E). These findings suggested T deficiency further attenuates mitochondrial biogenesis in the hippocampus of male APP/PS1 mice.

### Testosterone deficiency aggravates the imbalanced mitochondrial dynamics in the hippocampus of male APP/PS1 mice

**FIGURE 6:**
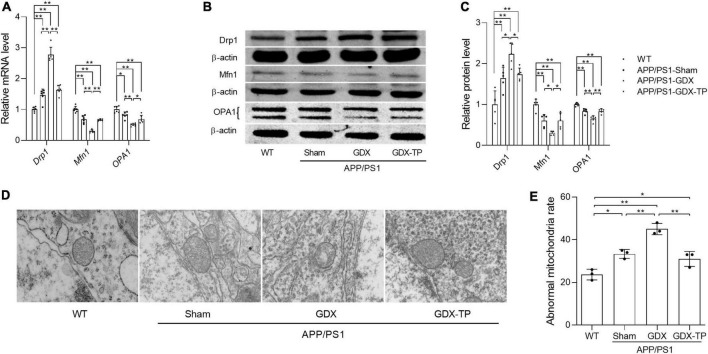
Effects of testosterone deficiency on mitochondrial dynamics genes and mitochondrial structure in the hippocampus of male APP/PS1 mice. **(A)** Relative mRNA levels of *Drp1*, *Mfn1*, and *OPA1* were detected by qPCR. **(B)** Representative blots for Drp1, Mfn1, and OPA1; **(C)** relative protein levels of Drp1, Mfn1, and OPA1 were analyzed by Western blot. The complete Western blots for each sample are provided in Supplementary Data. **(D)** Mitochondrial ultrastructure images were examined by transmission electron microscope. **(E)** The abnormal mitochondria rate was calculated by the number of abnormal mitochondria/total number of mitochondria. Data are expressed as the mean ± SD (*n* = 6 for qPCR, *n* = 5 for Western blot, *n* = 3 for electron microscope). *P < 0.05 and **P < 0.01.

Mitochondrial dynamics are also one of the approaches maintaining normal mitochondrial content. Mitochondria, as dynamic cell organelles, switch morphology between fragmented and tubular shapes via mitochondrial fission and fusion depending on different situations (Piquereau et al., 2013). Mitochondrial dynamics are regulated by the fission proteins Drp1 and Fis1, as well as the fusion proteins Mfn1, Mfn2, and OPA1 (Piquereau et al., 2013). Abnormal mitochondrial dynamics are involved in age-related neurodegenerative conditions. Therefore, the effects of T deficiency on the mitochondrial dynamics in the hippocampus of APP/PS1 mice were analyzed by detecting the altered expression levels of Drp1, Mfn1, and OPA1, which are involved in regulating mitochondrial dynamics. Relative to WT mice, Drp1 expression levels of male APP/PS1-Sham mice were significantly increased in the hippocampus (Figures 6A–C). Orchiectomy to APP/PS1 mice resulted in the further increased Drp1 expression levels in the hippocampus and TP supplementation restored Drp1 expression levels of APP/PS1-GDX mice to Sham mice level. In contrast, Mfn1 and OPA1 expression levels were significantly decreased in the hippocampus of APP/PS1-Sham mice compared with WT mice (Figures 6A–C). Castration to male APP/PS1 mice further reduced the Mfn1 and OPA1 expression levels in the hippocampus. In addition, mitochondrial ultrastructure displayed striking differences among the experimental groups (Figures 6D, E). Compared with mitochondria from the hippocampus of WT mice, which presented a normal mitochondrial structure with clear cristae, some mitochondria from the APP/PS1-Sham mice showed slightly disorganized cristae. Orchiectomy aggravated disorganized cristae of mitochondria from hippocampus of APP/PS1 mice and increased abnormal mitochondrial number. Testosterone supplementation to APP/PS1-GDX mice improved the ultrastructural alterations of mitochondrial cristae. The data above demonstrated that testosterone deficiency aggravates the imbalance of mitochondrial dynamics of male APP/PS1 mice, escalating mitochondrial fission in the hippocampus.

### Flutamide reduces the protective effects of testosterone on SH-SY5Y cells against H_2_O_2_-induced oxidative damage and cell death

**FIGURE 7:**
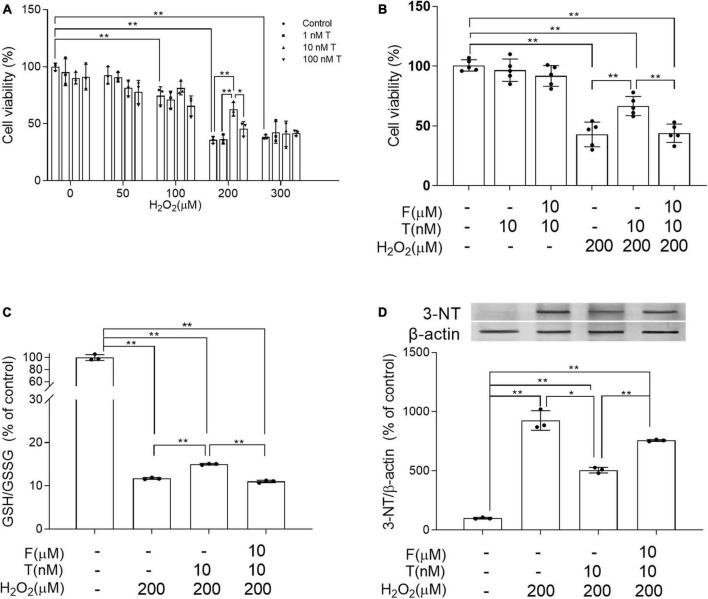
Effects of testosterone pretreatment on H_2_O_2_-induced oxidative damage and cell death in SH-SY5Y cells via androgen receptor. **(A)** Cell viability of SH-SY5Y cells exposed to 0–300 μM H_2_O_2_ for 24 h following 24 h pretreatment of 0–100 nM T. **(B)** Cell viability of SH-SY5Y cells was detected after cells being exposed to 10 μM F for 1 h, 10 nM T for 24 h or 200 μM H_2_O_2_ for 24 h. **(C)** The ratio of GSH/GSSG was assessed by spectrophotometry. **(D)** Representative blots for 3-NT and relative protein levels of 3-NT were analyzed by Western blot. The complete representative Western blots for each sample are provided in Supplementary Data. T, testosterone; F, flutamide. Data are expressed as the mean ± SD (*n* = 3–5 independent experiments). *P < 0.05 and **P < 0.01.

To study how T exerted its effects on neural cells, the alteration of oxidative stress was first investigated in human neuroblastoma SH-SY5Y cells due to critical involvement of oxidative stress in AD process. SH-SY5Y cells were exposed to 0–300 μM H_2_O_2_ for 24 h following 24 h pretreatment of 0–100 nM T. Appropriate concentration of T and H_2_O_2_ was chosen based on cell viability. Cell viability was significantly higher in 10 nM T + 200 μM H_2_O_2_ group than in 200 μM H_2_O_2_ group as well as 100 nM T + 200 μM H_2_O_2_ group (Figure 7A). Thus, 10 nM T and 200 μM H_2_O_2_ were used for later experiment. In addition to decreased cell viability, 200 μM H_2_O_2_ reduced GSH/GSSG ratio and increased 3-NT levels of SH-SY5Y cells. To validate whether the protective effects of T were mediated through androgen pathway, a specific AR antagonist flutamide was utilized. Administration of 10 μM AR antagonist flutamide for 1 h before T pretreatment inhibited the beneficial effects of 10 nM T on SH-SY5Y in cell viability, GSH/GSSG ratio and 3-NT levels following 200 μM H_2_O_2_ exposure (Figures 7B–D). Testosterone ameliorates H_2_O_2_-induced oxidative damage via androgen receptor.

### Flutamide attenuates the ameliorative effects of testosterone on H_2_O_2_-induced mitochondrial dysfunction of SH-SY5Y cells

**FIGURE 8:**
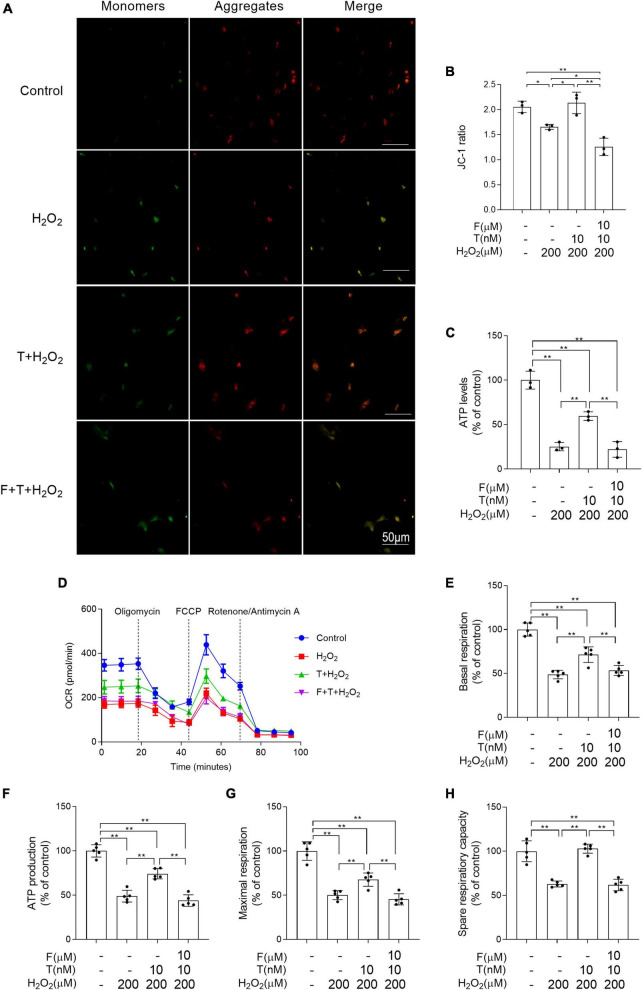
Effects of testosterone pretreatment on H_2_O_2_-induced mitochondrial dysfunction in SH-SY5Y cells via androgen receptor. **(A,B)** Mitochondrial membrane potential was detected by two-photon microscope. **(C)** ATP levels were assessed by spectrophotometry. **(D)** OCR was measured by XF24 Analyzer simultaneously before and after the sequential injection of oligomycin, FCCP, and rotenone/antimycin A. **(E)** Basal respiration, **(F)** ATP production, **(G)** maximal respiration, and **(H)** spare respiratory capacity were calculated sequentially. T, testosterone; F, flutamide. Data are expressed as the mean ± SD (*n* = 3–5 independent experiments). *P < 0.05 and **P < 0.01.

The mitochondrion is the main source of ROS and its dysfunction leads to oxidative stress. Thus, the following study was focused on mitochondrial function. Mitochondrial membrane potential and ATP levels in H_2_O_2_ group were significantly lower than that in control group and decreased mitochondrial membrane potential and ATP levels in H_2_O_2_ group were prevented via T pretreatment. Administration of flutamide before T pretreatment inhibited the above effects of T pretreatment on mitochondrial membrane potential and ATP levels of SH-SY5Y cells following H_2_O_2_ exposure (Figures 8A–C). By measuring the OCR (Figure 8D), it was discovered that the basal respiratory function, ATP production, maximal respiration and spare respiration capacity were decreased in H_2_O_2_ group relative to control group. T + H_2_O_2_ group showed higher basal respiratory function, ATP production, maximal respiration and spare respiration capacity than H_2_O_2_ group (Figures 8E–H). There is no significant difference between F + T + H_2_O_2_ group and H_2_O_2_ group in the basal respiratory function, ATP production, maximal respiration, and spare respiration capacity. Testosterone pretreatment alleviates H_2_O_2_-induced mitochondrial dysfunction of SH-SY5Y cells via androgen receptor.

### Flutamide decreases the efficacy of testosterone in protecting the mitochondrial biogenesis of SH-SY5Y cells from H_2_O_2_-damage

**FIGURE 9:**
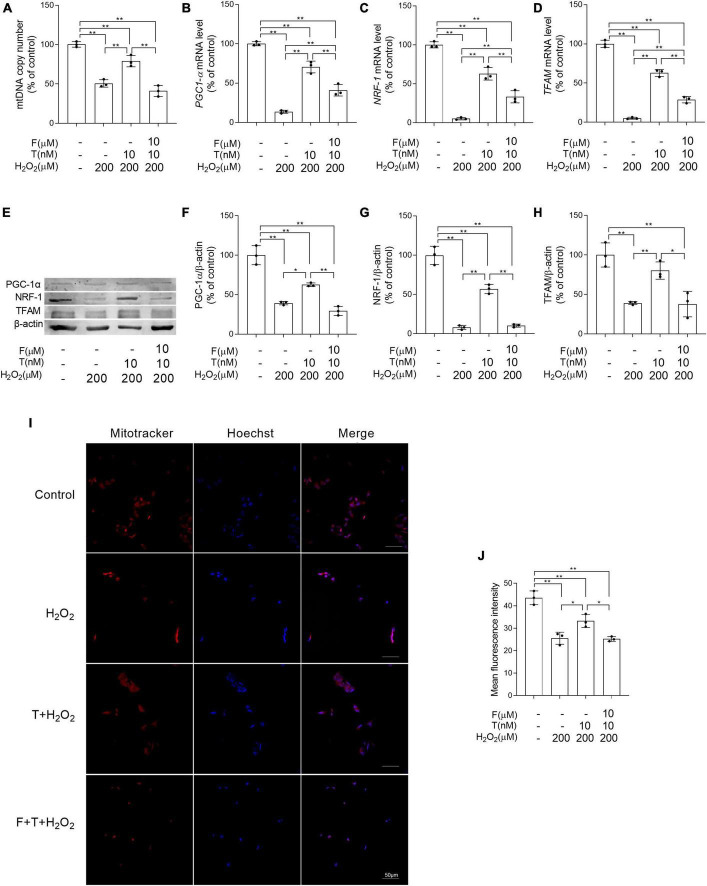
Effects of testosterone pretreatment on H_2_O_2_-damaged mitochondrial biogenesis in SH-SY5Y cells via androgen receptor. **(A)** mtDNA copy number, as well as **(B)** *PGC-1*α, **(C)** *NRF-1*, and **(D)** *TFAM* relative mRNA levels were detected by qPCR. **(E)** Representative blots of PGC-1α, NRF-1, and TFAM; relative protein levels of **(F)** PGC-1α, **(G)** NRF-1, and **(H)** TFAM were analyzed by Western blot. The complete Western blots for each sample are provided in Supplementary Data. **(I)** SH-SY5Y cells labeled with MitoTracker and Hoechst 33258 were examined by laser confocal microscopy. **(J)** Mitochondrial mass was expressed by the mean fluorescence intensity of mitochondria stained with MitoTracker. T, testosterone; F, flutamide. Data are expressed as the mean ± SD (*n* = 3 independent experiments). *P < 0.05 and **P < 0.01.

Some important parameters related to mitochondrial biogenesis were analyzed to evaluate the effects of T pretreatment on mitochondrial biogenesis under the condition of oxidative stress. It is found that decreased the mitochondrial copy number, as well as PGC-1α, NRF-1, and TFAM at mRNA and protein level was present in SH-SY5Y cells of H_2_O_2_ group compared with control group, while increased the mitochondrial copy number, as well as PGC-1α, NRF-1, and TFAM in SH-SY5Y cells was shown in T + H_2_O_2_ group relative to H_2_O_2_ group. No obvious difference in the mitochondrial copy number, as well as the expression of PGC-1α, NRF-1, and TFAM was detected in SH-SY5Y cells between F + T + H_2_O_2_ group and H_2_O_2_ group (Figures 9A–H). Moreover, mitochondrial mass (a parameter reflecting mitochondrial biogenesis to some extent) was analyzed via quantifying fluorescence intensity of MitoTracker Red by laser confocal microscopy. The mean fluorescence intensity in H_2_O_2_ group was significantly weaker than that in control group. T + H_2_O_2_ group presented strong mean fluorescence intensity relative to H_2_O_2_ group. There was no significant difference in mean fluorescence intensity between F + T + H_2_O_2_ group and H_2_O_2_ group (Figures 9I, J). Testosterone reduces H_2_O_2_-damage to mitochondrial biogenesis of SH-SY5Y cells via androgen receptor.

### Testosterone pretreatment alleviates H_2_O_2_-induced mitochondrial fragmentation of SH-SY5Y cells.

**FIGURE 10:**
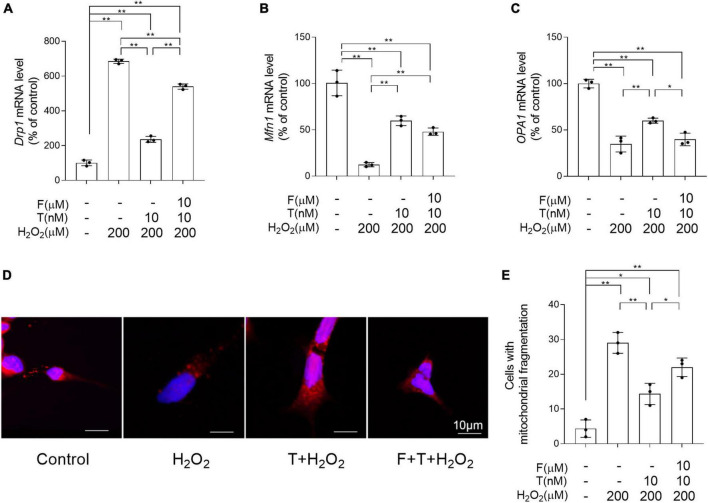
Effects of testosterone pretreatment on H_2_O_2_-damaged mitochondrial fragmentation in SH-SY5Y cells via androgen receptor. **(A)** *Drp1*, **(B)** *Mfn1*, and **(C)** *OPA1* relative mRNA levels were detected by qPCR. Mitochondria in the SH-SY5Y cells was labeled by MitoTracker and examined by laser confocal microscopy. Punctiform mitochondria were considered fragmented mitochondria, mitochondria with clear networks were considered tubular mitochondria. **(D)** Representative images of mitochondrial morphology stained by MitoTracker. **(E)** The number of cells was counted when 80% of mitochondria in the cell are under mitochondrial fragmentation. T, testosterone; F, flutamide. Data are expressed as the mean ± SD (*n* = 3 independent experiments). *P < 0.05 and **P < 0.01.

To determine the effects of T pretreatment on the balance of mitochondrial dynamics under the condition of oxidative stress, the altered mRNA levels of *Drp1*, *Mfn1*, and *OPA1* regulating mitochondrial dynamics were detected and the mitochondrial fragmentation extent was analyzed by MitoTracker Red CMXRos staining. Relative to control group, the significantly increased *Drp1*, as well as decreased *Mfn1* and *OPA1* was found in SH-SY5Y cells of H_2_O_2_ group (Figures 10A–C). On the contrary, compared with SH-SY5Y cells of H_2_O_2_ group, SH-SY5Y cells of T + H_2_O_2_ group showed the decreased *Drp1*, as well as the increased *Mfn1* and *OPA1*. For *Drp1*, SH-SY5Y cells demonstrated higher mRNA levels in F + T + H_2_O_2_ group than T + H_2_O_2_ group and lower mRNA levels in F + T + H_2_O_2_ group than H_2_O_2_ group. There is no significant difference of mRNA levels between F + T + H_2_O_2_ group and T + H_2_O_2_ group for *Mfn1*, as well as between F + T + H_2_O_2_ group and H_2_O_2_ group for *OPA1*. Furthermore, H_2_O_2_ group showed more SH-SY5Y cells with short punctiform mitochondria labeled by MitoTracker Red CMXRos dye than control group (Figures 10D, E). SH-SY5Y cells of T + H_2_O_2_ group had less short punctiform mitochondria and more long tubular mitochondria than SH-SY5Y cells of H_2_O_2_ group. No significant difference was found in the numbers of SH-SY5Y cells with short punctiform mitochondria between F + T + H_2_O_2_ group and H_2_O_2_ group, though the reduced trend in the numbers of SH-SY5Y cells with short punctiform mitochondria was detected in F + T + H_2_O_2_ group relative to H_2_O_2_ group. It was demonstrated that testosterone pretreatment reduces H_2_O_2_-induced mitochondrial fission, alleviating mitochondrial fragmentation of SH-SY5Y cells.

## Discussion

This study demonstrated that T deficiency exacerbates the mitochondrial dysfunction in the hippocampus of male APP/PS1 mice, a common animal model for AD. In addition to reducing mitochondrial content, T deficiency leads to the further level reduction in mitochondrial biogenesis related proteins, and the aggravation of dysregulated expression in mitochondrial dynamics proteins in the hippocampus of APP/PS1 mice. *In vitro* studies using SH-SY5Y cells validate T’s protective effects on the H_2_O_2_-induced mitochondrial dysfunction and expression alteration of the proteins related to mitochondrial biogenesis and mitochondrial dynamics. Thus, appropriate T levels might be beneficial for preventing/treating AD.

Testosterone, the gonadal hormone, has been reported for its neuroprotective effects on the many brain regions (Snyder et al., 2018), including the hippocampus. The previous studies showed beneficial effects of T on cognition in healthy aging men, in AD patients and in animals (Muller et al., 2008; Benice and Raber, 2009; Tan et al., 2019). Age-related T decline is associated with cognitive impairment of AD in older men (Muller et al., 2008). Elevated levels of Aβ were observed in AD men with low levels of T, indicating that T levels are inversely correlated to Aβ levels (Vegeto et al., 2020). Triple transgenic AD model mice with orchiectomy exhibit impaired cognitive performances and elevated levels of Aβ in the hippocampus compared to sham operated AD model mice (Rosario et al., 2006). Treatment with T or its metabolite dihydrotestosterone significantly attenuates AD pathological changes (Rosario et al., 2006, 2012; Vegeto et al., 2020). Similarly, the present study revealed that 4-month-old APP/PS1 mice behave spatial memory deficits, and that orchiectomy to them induces more severe cognitive deficits and synaptic protein diminution, as well as Aβ accumulation. Thus, T deficiency has an aggravating impact on the cognitive deficits of male APP/PS1 mice, as well as the AD pathological changes in the hippocampus of them.

Previous studies revealed the close relationship between T levels and antioxidative capability (Meydan et al., 2010; Cunningham et al., 2014; Torrens-Mas et al., 2020). T levels show positive relationship with the levels of the antioxidant glutathione S-transferase in men over 60 years old (Cunningham et al., 2014) and castration to adult male rats increases MDA levels and decreases SOD activity in the hippocampus (Meydan et al., 2010). In the present study, we also found orchiectomy further impairs hippocampal antioxidative system, reflected by increased levels of MDA and 3-NT, decreased ratio of GSH/GSSG, as well as attenuated activity of Mn-SOD and CuZn-SOD, in the hippocampus of the male APP/PS1 mice relative to APP/PS1-Sham group. The present finding suggested that T deficiency worsens the oxidative damage to the hippocampus of male APP/PS1 mice. The previous experimental data indicated a strong association between abnormal mitochondria and AD as well as AD animal model (Kandimalla et al., 2018; Manczak et al., 2018; Liu, 2022; Venkataraman et al., 2022). Twelfth-month-old APP transgenic mice, in addition to Aβ accumulation, show abnormal mitochondrial structure and function (Manczak et al., 2018), and even in the early-stage of AD, the patients already have mitochondrial dysfunction revealed by positron emission tomography (Venkataraman et al., 2022). In the present study, similar mitochondrial changes were observed in 4-month-old APP/PS1 mice, even in the absence of detectable Aβ accumulation. *In vitro* studies revealed that T improves mitochondrial function by increasing mitochondrial membrane potential (Grimm et al., 2014) and exerting protective effects against Aβ-induced mitochondrial deficits (Fisher et al., 2018). However, T deficiency by gonadectomy to 3-month-old male APP/PS1 mice induces further significant decrease in mitochondrial membrane potential and ATP levels, as well as complex IV activity relative to APP/PS1-Sham mice, indicating that T deficiency exacerbates mitochondrial dysfunction in male APP/PS1 mice.

Mitochondrial dysfunction precedes the AD pathology, and is critically involved in the pathogenesis of AD (Ashleigh et al., 2023). Normally, cells maintain healthy mitochondria through some processes such as mitochondrial biogenesis and mitochondrial dynamics for normal mitochondrial function. Mitochondrial biogenesis and mitochondrial dynamics act as important approaches to maintain mitochondrial homeostasis. However, under the pathological condition, mitochondrial biogenesis and mitochondrial dynamics are impaired, such as in AD patients and animal model (Kandimalla et al., 2018; Manczak et al., 2018; Li et al., 2022). APP transgenic mice show the decreased mitochondrial biogenesis related protein levels (Manczak et al., 2018), increased mitochondrial fission protein levels and reduced mitochondrial fusion protein levels in the hippocampus (Wang et al., 2008, 2009; Reddy et al., 2011). Similarly, the present study detected the diminished mitochondrial biogenesis related protein levels (PGC-1α, NRF-1, and TFAM), the reduced mitochondrial content (CS activity and mtDNA copy number), as well as increased mitochondrial fission protein levels (Drp1) and reduced mitochondrial fusion protein levels (Mfn1 and OPA1) in the hippocampus of APP/PS1 mice, showing attenuated mitochondrial biogenesis and imbalanced mitochondrial dynamics (increased mitochondrial fission over mitochondrial fusion) in this kind of AD animal model. The previous studies showed that gonadectomy triggers the decreased expression of mtDNA gene in hippocampus of adult male mice, which might be due to the downregulation of mitochondrial biogenesis related proteins (Pronsato et al., 2020; Ren et al., 2022). In the present study, orchiectomy-APP/PS1 mice exhibits further reduction in the levels of mitochondrial biogenesis related proteins, mitochondrial content, and mitochondrial fusion proteins, as well as increment in the levels of mitochondrial fission protein, compared to APP/PS1-Sham mice. The previous study showed that excessive mitochondrial fission leads to mitochondrial fragmentation, a prototypical feature of neurodegeneration (Knott et al., 2008; Reddy et al., 2011). Ultrastructurally, orchidectomy-APP/PS1 mice show the increased number of abnormal mitochondria with disorganized cristae in the hippocampus compared with APP/PS1-Sham mice. It was demonstrated that T deficiency of male APP/PS1 mice aggravates hippocampal mitochondrial biogenesis impairment and promotes mitochondrial fragmentation.

The findings above suggested that T deficiency exert important effects on compromised mitochondrial function of male APP/PS1 mice. In the experiments using SH-SY5Y cells, we found the protective effects of T on the H_2_O_2_-induced mitochondrial dysfunction and expression alteration of the proteins involved in mitochondrial biogenesis/mitochondrial dynamics. Administration of AR antagonist flutamide significantly weakens the beneficial effects of T pretreatment on the relative parameters analyzed in H_2_O_2_-treated SH-SY5Y cells, demonstrating a critical role of classical AR pathway in maintaining mitochondrial function. Previous studies showed the presence of potential AR binding sites in the *TFAM* promoter (Liu et al., 2019), and *Drp1* promoter (Choudhary et al., 2011) in none-neural cells, indicating that androgen is directly involved in regulating mitochondrial biogenesis and mitochondrial dynamics. It was presumed that cytoplasmic androgen-AR complex enters nucleus and binds directly to AR-binding site in the promoter region of *TFAM*/*Drp1* gene to regulate the expression levels of them, and further influencing the mitochondrial function through mitochondrial biogenesis and mitochondrial dynamics. In AD with low T level or T deficiency, decreased signals might occur to AR binding site of gene promoter region regulating mitochondrial function. Thus, the altered expression of TFAM and Drp1 in T-deficiency male APP/PS1 mice demonstrates the impaired mitochondrial biogenesis aggravation and escalated mitochondrial fission relative to APP/PS1-Sham mice, leading to an overall reduction of new mitochondria, an increased number of fragmented mitochondria, and an accumulation of defective mitochondria in the hippocampus. These alterations contribute to increment of ROS production and promotion of the hippocampal pathological alteration in T-deficiency male APP/PS1 mice, such as deposition of Aβ.

However, it is worth noting that there are some problems unresolved in the present study. Our analysis about the influence of androgen upon mitochondrial biogenesis/mitochondrial dynamics was based on the presence of potential AR binding sites in *TFAM*/*Drp1* gene promoter region of non-neural cells, instead of neurons (Choudhary et al., 2011; Liu et al., 2019). Whether direct regulation of androgen to mitochondrial biogenesis/mitochondrial dynamics is also present in neural cells was not verified yet in the present studies. Moreover, considering that T deficiency also induces the altered levels of PGC-1α, NRF-1, Mfn1, and OPA1 in the hippocampus of male APP/PS1 mice, it is unknown whether their altered expression levels are directly affected by androgen as TFAM/Drp1 is or just the consequence of altered TFAM/Drp1 expression induced by T deficiency. It is necessary in the nervous tissue to explore whether AR binding sites are present in the promoter region of these genes regulating mitochondrial biogenesis/mitochondrial dynamics in the future studies. In addition, only the hippocampus of APP/PS1 mice at 4 months old was chosen for observation in the present study. It is also highly valuable to search for similar phenotypes in other brain regions beyond the hippocampus, across diverse sex-specific models of AD and in individuals with different T levels due to varying ages. This extensive exploration and comparison will provide a more comprehensive understanding of AD and its progression.

## Conclusion

**FIGURE 11:**
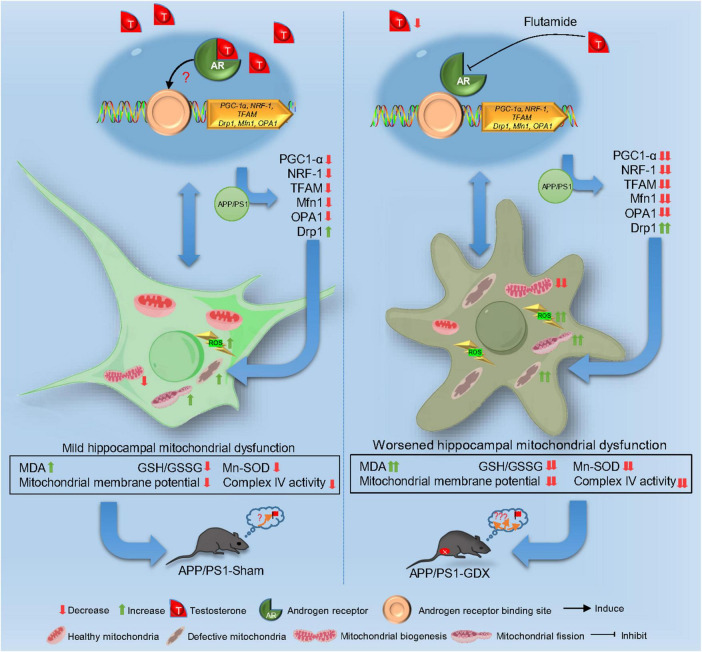
Schematic image.

Altogether, T deficiency exacerbates mitochondrial dysfunction, intensifying oxidative damage and AD pathological alteration in male APP/PS1 mice. T-deficiency-induced aggravation in the impaired mitochondrial biogenesis and imbalanced mitochondrial dynamics might underlie mitochondrial dysfunction in the hippocampus of gonadectomized male APP/PS1 mice to a certain extent. The aggravation of impaired mitochondrial biogenesis and escalated mitochondrial fission lead to the reduced mitochondrial renewal, increased fragmented mitochondria and subsequent accumulated defective mitochondria in the hippocampus, thus, promoting disease progression of AD. Appropriate T levels in the early stage of AD might be beneficial in delaying AD pathology by improving mitochondrial biogenesis and mitochondrial dynamics (Figure 11).

## Data availability statement

The raw data supporting the conclusions of this article will be made available by the authors, without undue reservation.

## Ethics statement

The animal study was approved by the Laboratory Animal Ethical and Welfare Committee of Hebei Medical University. The study was conducted in accordance with the local legislation and institutional requirements.

## Author contributions

TZ: Formal analysis, Funding acquisition, Investigation, Resources, Validation, Visualization, Writing – original draft. YC: Investigation, Writing – review & editing. YueW: Investigation, Writing – review & editing. YuW: Investigation, Writing – review & editing. JW: Investigation, Writing – review & editing. XJ: Investigation, Resources, Writing – review & editing. GZ: Data curation, Formal analysis, Funding acquisition, Writing – review & editing. GS: Conceptualization, Funding acquisition, Supervision, Visualization, Writing – review & editing. RC: Data curation, Formal analysis, Funding acquisition, Writing – review & editing. YK: Conceptualization, Data curation, Project administration, Supervision, Visualization, Writing – review & editing.
