# Supplementary material for: Testosterone deficiency worsens mitochondrial dysfunction in APP/PS1 mice
Source: Front Aging Neurosci. 2024 May 1;16:1390915. doi: 10.3389/fnagi.2024.1390915 (PMC11094339; doi:10.3389/fnagi.2024.1390915)
Supplement: Supplementary file 1 [file Data_Sheet_1.PDF]

Supplementary material

Electrophoretic gels and blots

Figure 2B

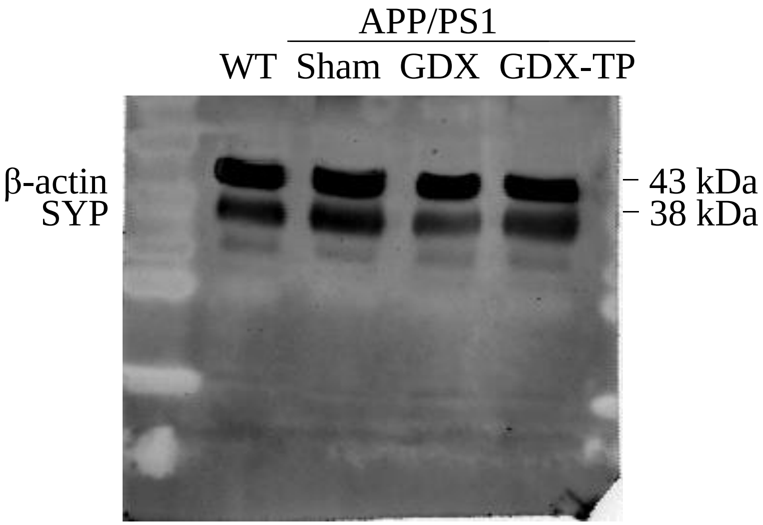

Figure 2D

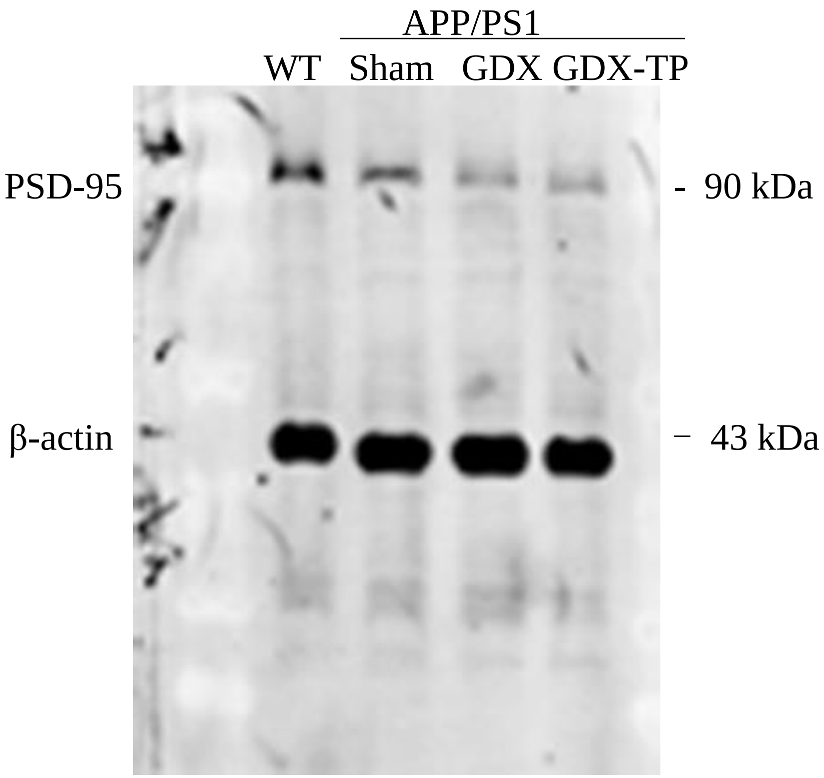

Figure 3G

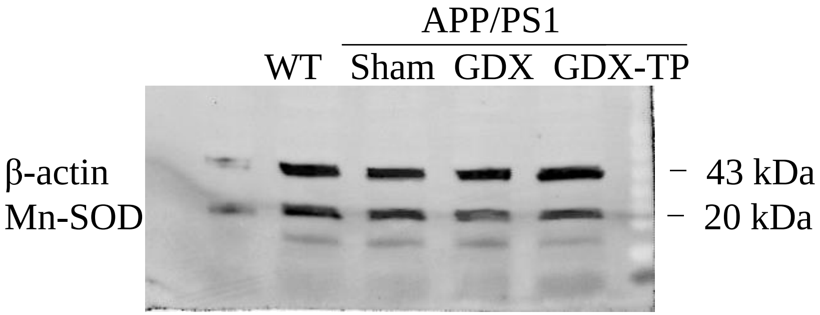

Figure 5D

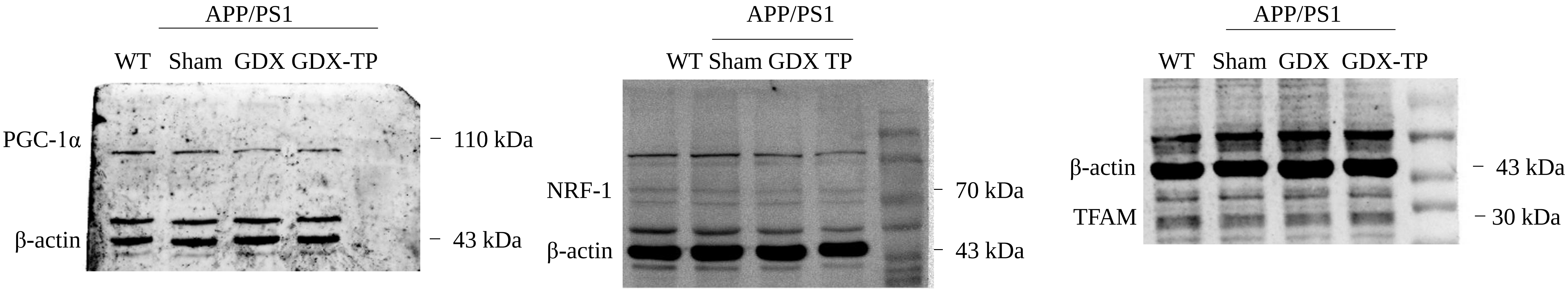
APP/PS1
WT Sham GDX GDX-TP
PGC-1α
β-actin
110 kDa
43 kDa
APP/PS1
WT Sham GDX TP
NRF-1
β-actin
70 kDa
43 kDa
APP/PS1
WT Sham GDX GDX-TP
β-actin
TFAM
43 kDa
30 kDa

Figure 6B

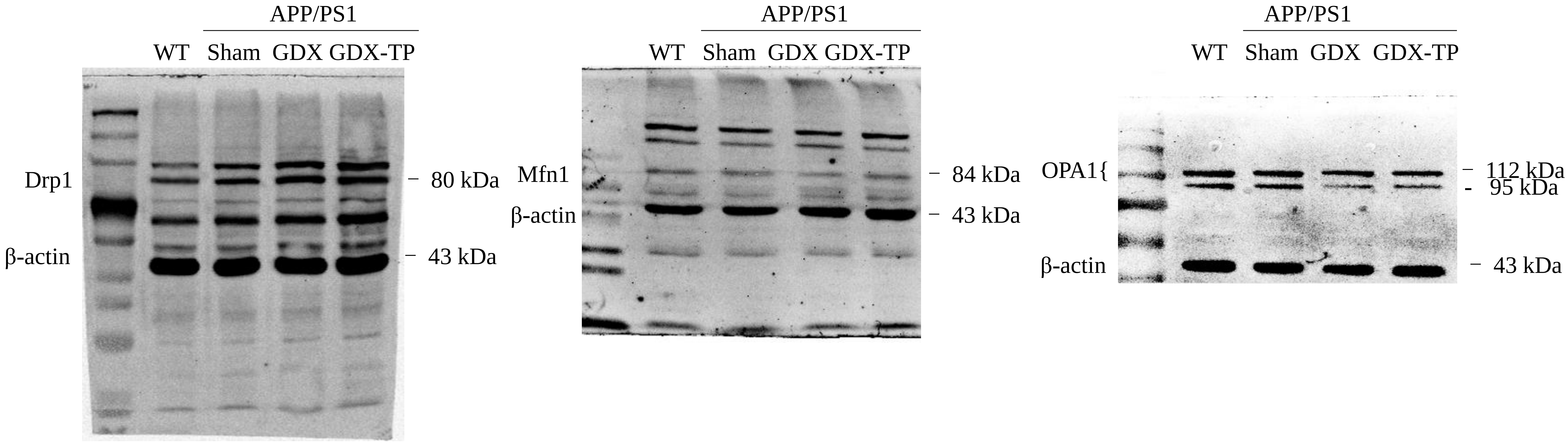
APP/PS1
WT
Sham
GDX
GDX-TP
Drp1
β-actin
80 kDa
43 kDa
APP/PS1
WT
Sham
GDX
GDX-TP
Mfn1
β-actin
84 kDa
43 kDa
APP/PS1
WT
Sham
GDX
GDX-TP
OPA1{
β-actin
112 kDa
95 kDa
43 kDa

Figure 7D

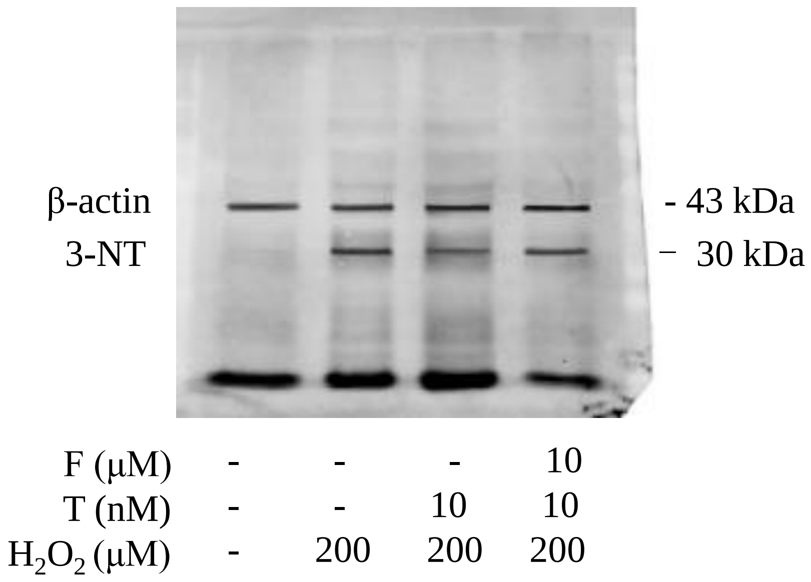

Figure 9E

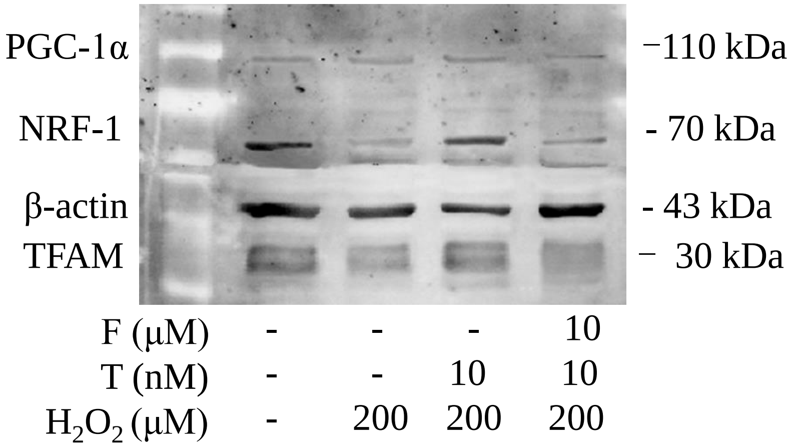
PGC-1α
NRF-1
β-actin
TFAM
−110 kDa
- 70 kDa
- 43 kDa
− 30 kDa
F (μM) - - - 10
T (nM) - - 10 10
H_2O_2 (μM) - 200 200 200
